# Commercial large language models for oral cavity cancer staging using descriptive pre-treatment MRI reports: ready for standalone use in clinical practice?

**DOI:** 10.1186/s13244-026-02409-y

**Published:** 2026-09-21

**Authors:** Qi Yong Hemis Ai, Hoi Ming Kwok, Ming-Yi Lu, Tracy T. S. Lau, Kuo Feng Hung, Lun M. Wong, Tiffany Y. So, Ann D. King, Ho Sang Leung

**Affiliations:** 1 Department of Imaging and Interventional Radiology, Faculty of Medicine, The Chinese University of Hong Kong, Shatin, Hong Kong SAR; 2 Department of Diagnostic and Interventional Radiology, Princess Margaret Hospital, Hospital Authority, Kowloon, Hong Kong SAR; 3 Department of Oral and Maxillofacial Surgery, Chung Shan Medical University Hospital, Taichung, Taiwan; 4 School of Dentistry, College of Oral Medicine, Chung Shan Medical University, Taichung, Taiwan; 5 Department of Oncology, Princess Margaret Hospital, Hospital Authority, Kowloon, Hong Kong SAR; 6 Oral and Maxillofacial Radiology, Applied Oral Sciences & Community Dental Care, Faculty of Dentistry, The University of Hong Kong, Pokfulam, Hong Kong SAR; 7 Department of Imaging and Interventional Radiology, Prince of Wales Hospital, Hospital Authority, Shatin, Hong Kong SAR

**Keywords:** Cancer staging, Magnetic resonance imaging, Oral cancer, Large language models, Multidisciplinary team

## Abstract

**Objectives:**

MRI is used for staging head and neck cancer (HNC), but assigning T- and N-category criteria requires specialised expertise, and so many institutions offer only descriptive MRI reports. This study assessed potentials of commercial large language models (LLMs) to stage oral cavity cancer (OCC) using descriptive MRI reports and compared their accuracy with human experts.

**Materials and methods:**

104 eligible MRI reports were processed by five commercial LLMs (ChatGPT5.4, ChatGPT5.0, ChatGPT4.1, Gemini3.1 and DeepSeekV3.2). T- and N-categories, and overall stage were extracted from outputs of the LLMs. MRI reports were also staged by two multidisciplinary team (MDT) members. Accuracies of the LLMs and MDT members for cancer staging were assessed against the reference staging standard and compared using the McNemar test.

**Results:**

The LLMs showed accuracy of 54.8–76.0% for T-categorisation, 63.5–88.5% for N-categorisation, and 53.8–80.8% for overall stage. Compared with DeepSeekV3.2, ChatGPT and Gemini3.1 showed significantly higher accuracy (*p* ≤ 0.001), except for Gemini3.1 for T-categorisation (*p* = 0.07). No differences in accuracy for staging between ChatGPT versions and between them and Gemini3.1 (*p* = 0.08 to > 0.99). MDT members showed accuracy of 74.0–76.9% for T-categorisation, 76.9–77.9% for N-categorisation and 68.3–71.2% for overall stage. The tested LLMs did not consistently outperform MDT members for staging.

**Conclusion:**

Variable tested LLM performance in staging OCC from descriptive MRI reports suggested that they are not suitable as standalone staging tools. Additionally, Low MDT staging accuracy highlighted the need for structured reports that include clinical cancer staging to facilitate MDT assessment, thus potentially ensuring optimised disease management.

**Key Points:**

- **Question** Can commercial LLMs accurately assign cancer stage based on descriptive MRI reports to overcome the lack of specialised expertise in clinical practice?
- **Findings** Tested LLMs demonstrated variable performances, ranging 54.8–76.0% for T-categorisation, 63.5–88.5% for N-categorisation and 53.8–80.8% for overall stage, and failed to consistently outperform MDT members.
- **Critical relevance statement** Variable tested LLM performance in staging OCC from descriptive MRI reports suggested that they are not suitable as standalone staging tools. MDT members’ low performances highlight that structured MRI reports with clinical cancer staging are needed to improve multidisciplinary assessment.

**Graphical Abstract:**

**Figure:**
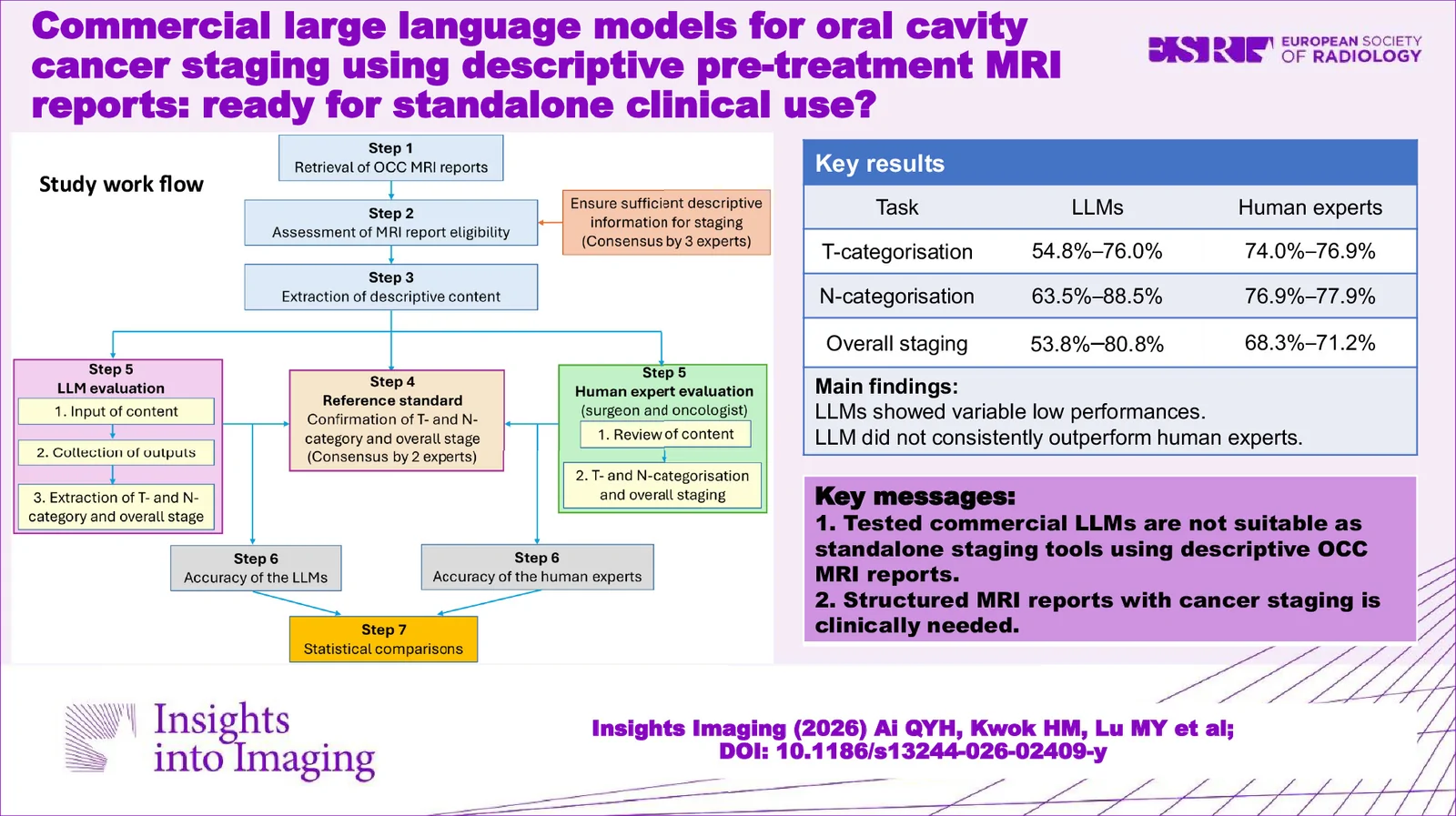

## Introduction

In head and neck cancer (HNC), pre-treatment cancer staging is essential for disease management [1]. It provides a critical reference for the design of treatment strategies, including the selection of surgical or radiotherapeutic approaches and the addition of chemotherapy [1]. It is also commonly used for the prediction of post-treatment outcomes and facilitates easy communication among multidisciplinary team (MDT) members, clinicians and patients.

Planning HNC treatment, both surgical and non-surgical, staging relies heavily on imaging using magnetic resonance imaging (MRI) and/or computed tomography (CT) of the head and neck to map the extent of the primary tumour and nodal metastases. The staging criteria in the American Joint Committee on Cancer (AJCC)/Union of International Cancer Committee (UICC) Cancer Staging Manuals vary between different head and neck regions, and the staging systems are updated periodically. For oral cavity cancer (OCC), criteria in the 8th edition for T-categorisation of primary tumours include tumour size, depth of invasion and extent of invasion, while the N-categorisation for nodal metastases includes laterality, size and number of nodes. This process is time-consuming and requires specialised radiology expertise, and so only descriptive MRI reports are often available without assigning T- and N-categories, which are major contributors to the overall stage [2].

In recent years, large language models (LLMs), which are artificial intelligence (AI)–based powerful language models, have been integrated into many aspects of healthcare management [3–6]. These models leverage deep learning techniques and vast amounts of textual data to generate human-like responses and perform complex reasoning. These LLMs could potentially provide T- and N-categories where only descriptive reports are available and, in the future, may be able to indicate when important information is missing [6–24]. In some studies where commercial LLMs such as ChatGPT and DeepSeek have been applied to head and neck imaging, they have shown promise in characterising tumours, planning treatment and predicting treatment outcome [15, 16, 23, 25–41]. However, few studies have investigated the accuracy of these commercial LLMs in assigning T- and N-categories and stage to the radiology report [6, 15, 16].

In this study, we aimed to investigate the potential utility of the commercial LLMs as diagnostic support tools by assessing the accuracy of ChatGPT, Gemini and DeepSeek in staging OCC based on the pre-treatment MRI reports that contained sufficient descriptive content of OCC for clinical T- and N-categories assignment and stage. Accuracies of the LLMs for T-categorisation, N-categorisation and overall staging were analysed and compared with those of an oncologist and an oral surgeon, who are members of the MDT for OCC management.

## Materials and methods

### Patients and MRI reports

**Fig. 1:**
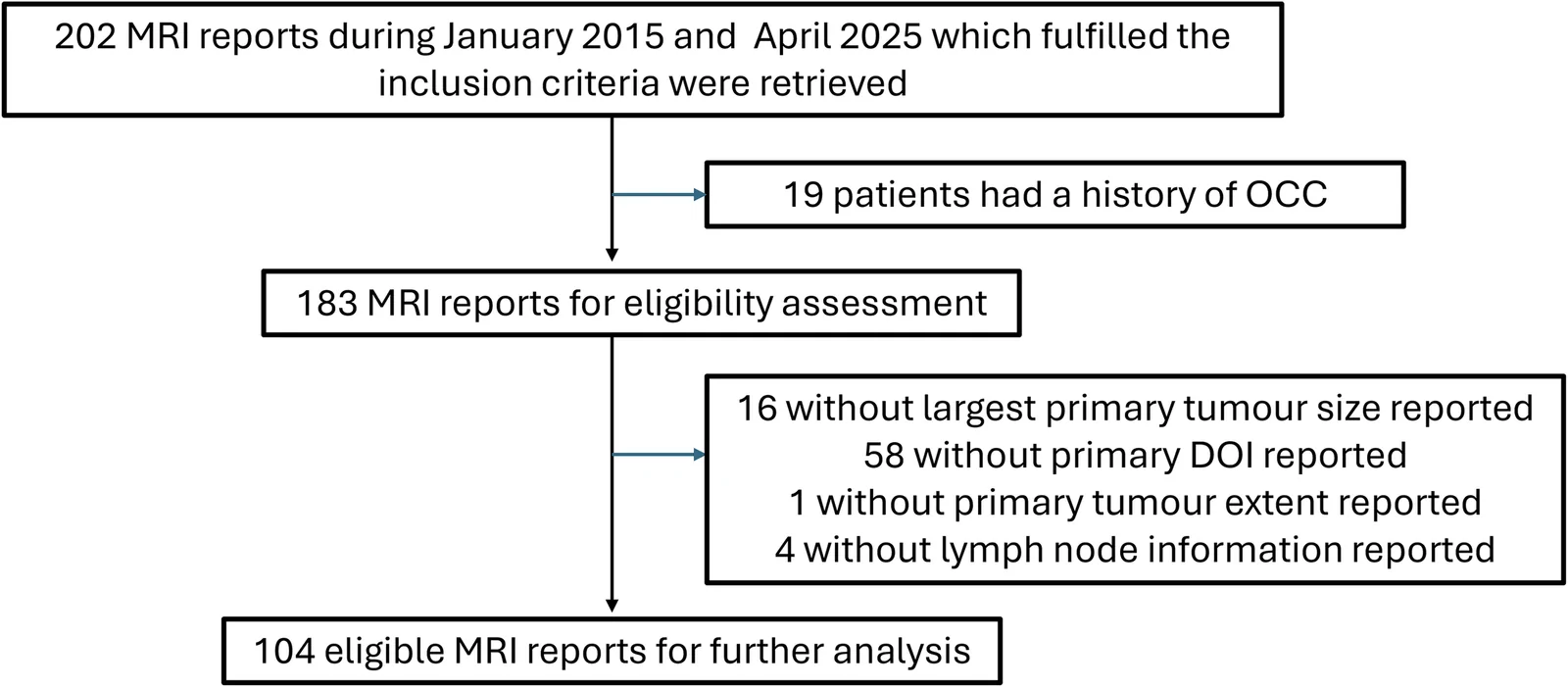
Flowchart of MRI report selection

This retrospective study was approved by the Institutional Review Board (Joint Chinese University of Hong Kong and New Territories East Cluster Clinical Research Ethical Committee) (Ref. No. 2025.567), and the requirement for written consent was waived for this study. All study procedures complied with the tenets of the Declaration of Helsinki. Details of the inclusion and exclusion criteria are shown in the supplementary file. 202 MRI reports between January 2015 and April 2025 were retrieved. After excluding 98 MRI reports, of which the majority were without radiological depth of invasion, which was later included in the staging criteria after 2017, 104 eligible MRI reports remained for further analysis (Fig. 1). Details of the pre-treatment MRI reports acquisition and eligibility analysis are shown in the supplementary file.

### Reference standard of the T-category, N-category and overall stage

The T- and N-categories and overall stage were assigned in consensus between two board-certified radiologists with subspecialty in head and neck radiology by discussion (H.S.L. and H.M.K.) according to the 8th edition of the AJCC/UICC Cancer Staging Manuals and a refinement to the criteria for staging OCC in 2020 [42–44] (Supplementary Table 1). The final assessment of T- and N-categories and overall stage was used as the reference standard for the evaluation of performances of the LLMs and MDT members.

### OCC staging by LLMs and data collection from the LLM outputs

This study was conducted using three versions of ChatGPT (ChatGPT5.4, ChatGPT5.0 and ChatGPT4.1), Gemini3.1 and DeepSeekV3.2 accessed via the websites. Details of the procedures are shown in the supplementary file. The first round of outputs from the LLMs was collected for further information assessment. The above procedure was repeated after a time interval of maximum 3 months to assess the intra-observer variability of the LLMs for OCC staging based on the MRI reports.

After collecting all the outputs, the following data were extracted from each of the outputs for analysis: (1) whether the referenced cancer staging manual was mentioned (yes or no), (2) staging criteria that were referred by LLMs, (3) T-category, (4) N-category and (5) overall stage. Only the final recommendations in clinical T- and N-categories and overall stages from the outputs were recorded, irrespective of whether the outputs included any hypothesised scenarios or involved uncertainty. Those outputs which did not clearly provide final recommendations in clinical cancer staging were recorded as “not given” in the study for further analysis.

### OCC staging by human experts (oncologist and oral surgeon)

The same descriptive content from each eligible MRI report was separately reviewed by an oral surgeon with 18-year experience in oral cancer surgery (M.Y.L.) and an oncologist with 13-year experience in head and neck cancer treatment (T.T.S.L.). Two human experts were only requested to provide the cancer staging for the OCC MRI reports according to their work experiences, without clarifying whether they were allowed to refer to any versions/editions of the cancer staging manuals and no time constraints were imposed during the staging process. The T- and N-categories and overall stage from two human experts were recorded for further analysis.

### Data processing and statistical analysis

All collected data were initially analysed descriptively to summarise the staging categories determined by LLMs and two human experts from MDT. For each MRI output, T-category, N-category and overall stage determined by LLMs were considered as “correct” if they exactly matched the corresponding reference standard (i.e., LLM-generated: T4a; and the reference standard: T4a); otherwise, they were classified as “incorrect” (i.e., LLM-generated: N2a; and the reference standard: N2c). Any missing outputs or hallucinated outputs were regarded as “incorrect” in the following analysis. The accuracy for T-categorisation, N-categorisation and overall staging was analysed separately. Furthermore, the macro-averaged F1-score, per-class precision and recall of the LLMs for T- categorisation, N-categorisation and overall staging were calculated. Details of the methods are shown in the supplementary file.

The accuracy for T-categorisation, N-categorisation and overall staging between two LLMs and between the LLMs and human experts was compared using the McNemar test if the number of discordant pairs was ≥ 25 or using exact McNemar test if the number of discordant pairs was < 25. Inter-observer agreements for T-categorisation, N-categorisation and overall staging between (1) the reference standard and the LLMs and human experts; (2) between two rounds of the testing for each of the LLMs; and (3) two human experts were evaluated using weighted Cohen’s kappa test and the coefficients were calculated. Statistical analyses were performed using SPSS version 26.0 (IBM, Armonk, NY, USA). All statistical tests were two-sided and a *p*-value of < 0.05 was used to indicate statistical significance, and Bonferroni correction to the significant *p*-value was performed if applicable.

## Results

### Patients and MRI reports

Patient demographics, primary tumour site, reference T- and N-categories and overall stage are shown in Table 1. The OCC originated from the oral tongue in over 65% patients, over 75% of patients had advanced primary disease (T3 or T4), over 50% of patients had advanced nodal disease (N2 or N3) and over 80% of patients were Stage III or IV, of which the majority were Stage IVa (Table 1).

**Table 1:** Demographics, primary tumour site, reference clinical T- and N-category and overall stage of 104 patients

	Number of patients (%) *N* = 104
Sex	
Female	43 (41.3%)
Male	61 (58.7%)
Age (years)	
Median (range)	67 (28–95)
Primary tumour location	
Oral tongue	68 (65.4%)
Retromolar trigone	30 (28.9%)
Gingiva	4 (3.8%)
Hard palate	2 (1.9%)
Clinical T-category (8th AJCC) (as reference standard)	
T1	4 (3.8%)
T2	18 (17.3%)
T3	22 (21.2%)
T4a	42 (40.4%)
T4b	18 (17.3%)
Clinical N-category (8th AJCC) (as reference standard)	
N0	44 (42.3%)
N1	10 (9.6%)
N2a	1 (0.9%)
N2b	27 (26.0%)
N2c	14 (13.5%)
N3a	0 (0%)
N3b	8 (7.7%)
Clinical overall stage (8th AJCC) (as reference standard)	
Stage I	4 (3.8%)
Stage II	15 (14.4%)
Stage III	12 (11.6%)
Stage IVa	50 (48.1%)
Stage IVb	23 (22.1%)

### Performance of LLMs in assigning T- and N-categories and overall stage based on descriptive OCC MRI reports

Among the LLMs, the reference staging manual was described in all of the outputs from the ChatGPT5.0 (100%), ChatGPT4.1 (100%), Gemini3.1 (100%), and in 97/104 (93.3%) outputs from the DeepSeekV3.2 and 85/104 (81.7%) outputs from the ChatGPT5.4. All these outputs, with the description of the referenced cancer staging manual, clarified that the staging was referred to the 8th edition of the AJCC Cancer Staging Manual.

**Fig. 2:**
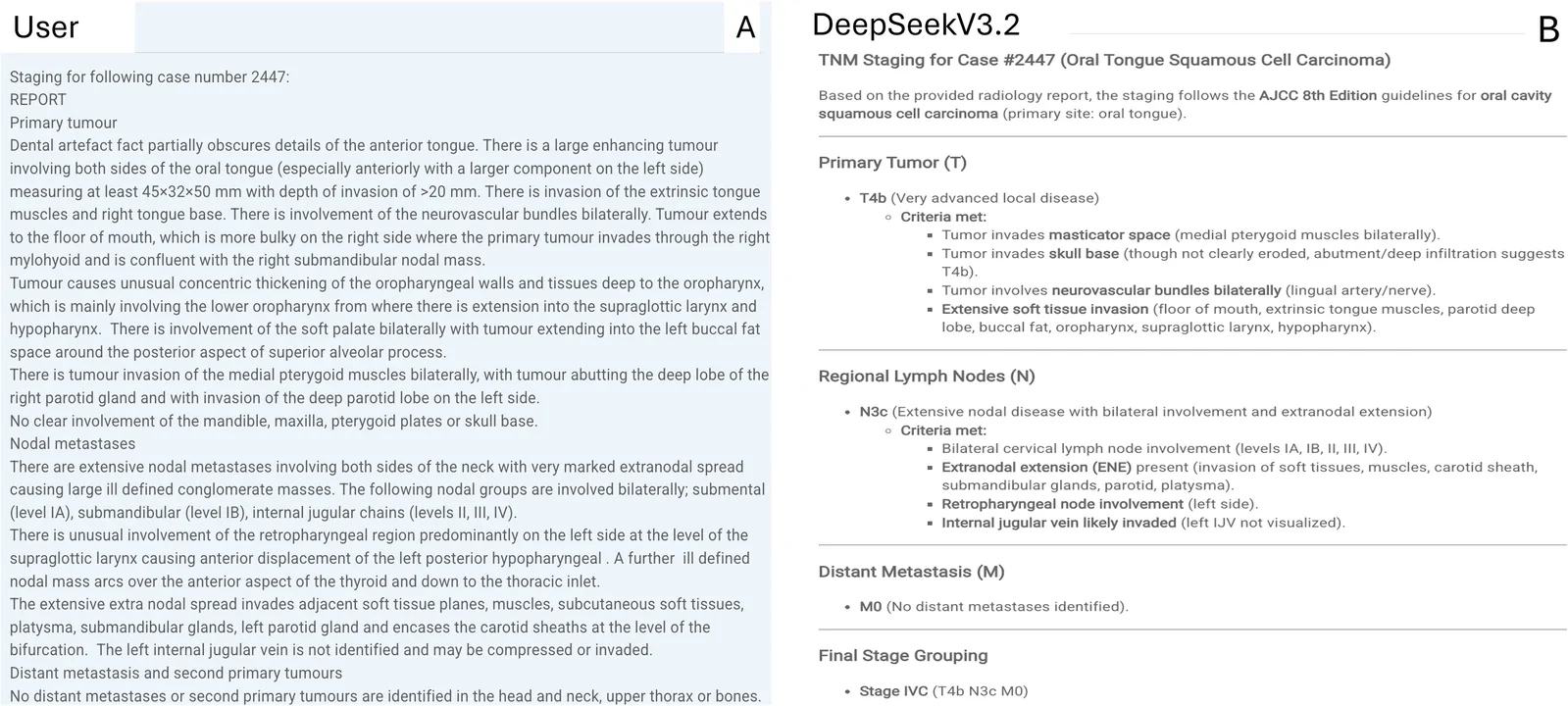
An example of hallucination from the large language model. An MRI report of oral cavity cancer was input to the DeepSeekV3.2 (**A**), with an output (**B**) showing a hallucinated N-category of N3c, which does not exist in the referenced manual (clarified as the 8th edition of AJCC Cancer Staging Manual in the output). Such hallucinations have the potential to mislead the LLM users, especially those who lack relevant knowledge

ChatGPT5.0 and Gemini3.1 provided T-category, N-category and overall stage in all of the outputs. ChatGPT5.4 did not provide overall stage in 1 output; ChatGPT4.1 did not provide N-category and overall stage in 2 and 1 outputs, respectively; and DeepSeekV3.2 did not provide T-category, N-category and overall stage in 2, 3 and 4 outputs, respectively. Details of the T- and N-categories and overall stage extracted from the outputs generated by ChatGPT, Gemini3.1 and DeepSeekV3.2 are shown in Table 2. DeepSeekV3.2 classified 1/104 reports as N3c based on the 8th edition of AJCC Cancer Staging Manual for OCC (Fig. 2).

**Table 2:** LLMs and human experts for OCC staging based on the descriptive MRI report

	Reference standard*	LLMs					Human experts	
		ChatGPT5.4	ChatGPT5.0	ChatGPT4.1	Gemini3.1	DeepSeekV3.2	Oral surgeon	Oncologist
Clinical T-category								
T1	4	4	4	6	4	10	9	8
T2	18	18	17	21	18	18	15	23
T3	22	38	19	18	46	20	22	15
T4a	42	31	50	46	18	48	37	43
T4b	18	13	14	13	18	6	21	15
Not provided	-	0	0	0	0	2	0	0
Clinical N-category								
N0	44	49	47	45	46	40	43	45
N1	10	7	8	12	7	28	13	14
N2a	1	2	2	0	1	1	4	0
N2b	27	24	23	23	25	20	23	30
N2c	14	15	13	13	13	10	8	13
N3a	0	0	0	0	0	0	0	0
N3b	8	7	11	9	12	1	13	2
N3c*	-	0	0	0	0	1*	0	0
Not provided	-	0	0	2	0	3	0	0
Clinical overall stage								
Stage I	4	4	4	6	4	8	8	8
Stage II	15	16	14	16	16	13	10	16
Stage III	12	22	12	13	21	18	19	9
Stage IVa	50	44	54	50	38	57	38	54
Stage IVb	23	17	20	18	25	4	29	17
Not provided	-	1	0	1	0	4	0	0

*LLM* larger language model, *OCC* oral cavity cancer

* N3c does not exist in the 8th edition of AJCC Cancer Staging Manual for OCC but reported by DeepSeekV3.2

**Fig. 3:**
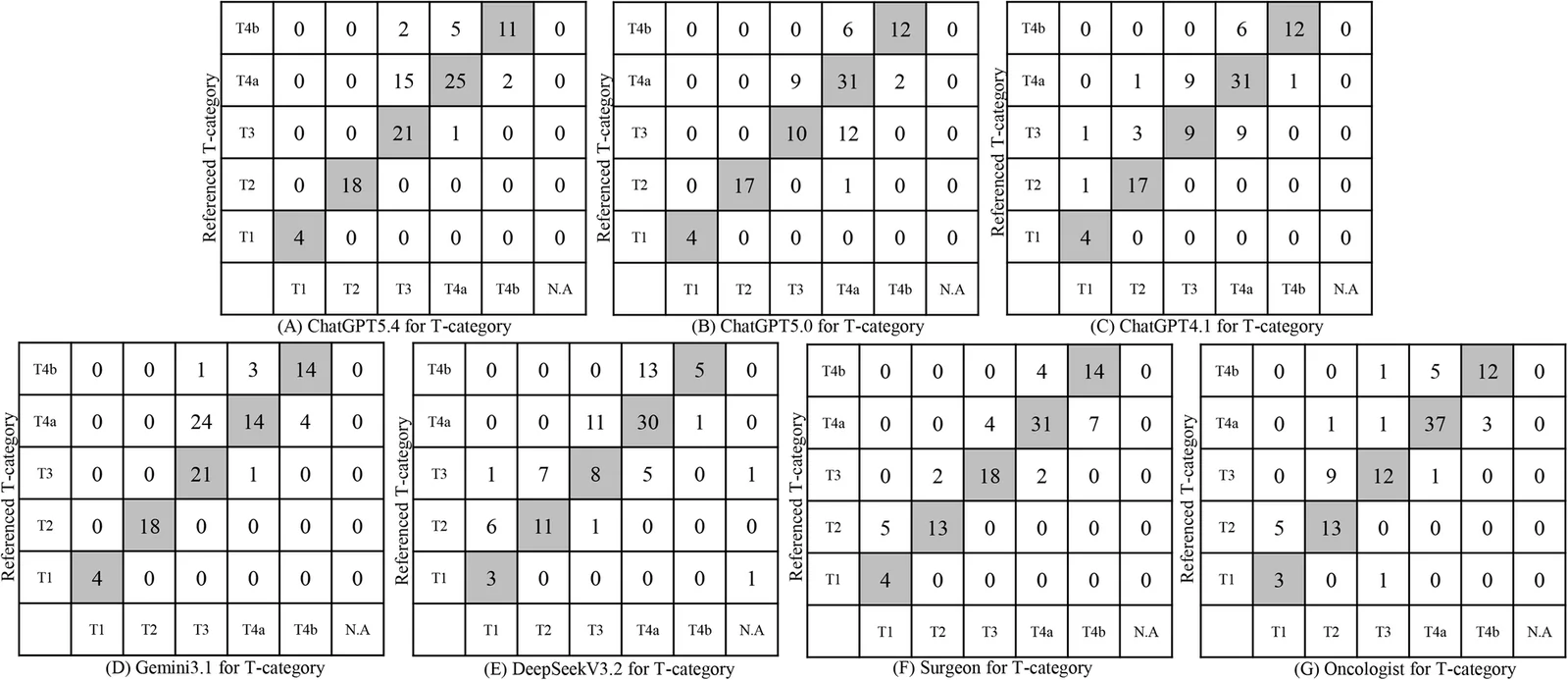
Confusion matrix for T-categorisation by five LLMs (**A**–**E**) and human experts (**F**, **G**)

**Fig. 4:**
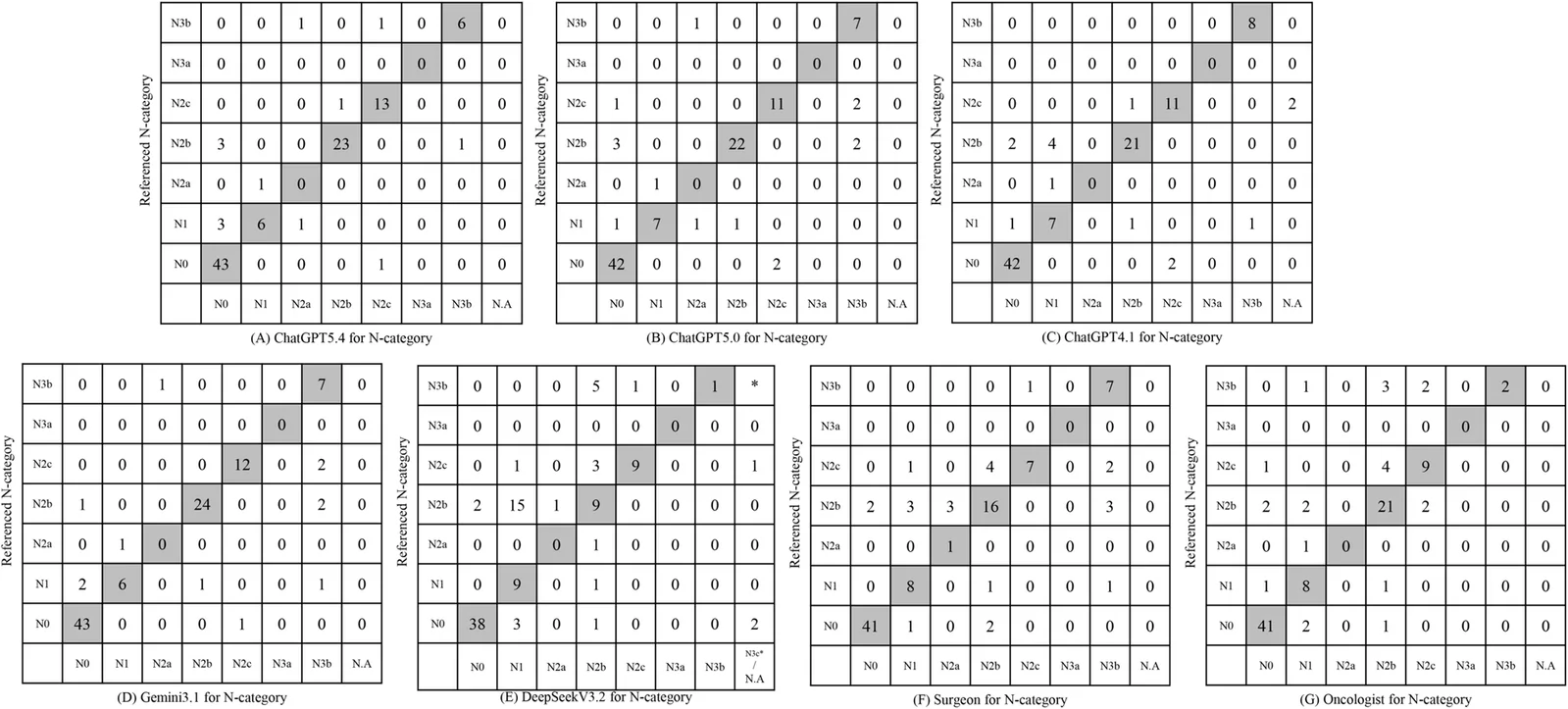
Confusion matrix for N-categorisation by LLMs (**A**–**E**) and human experts (**F**, **G**)

**Fig. 5:**
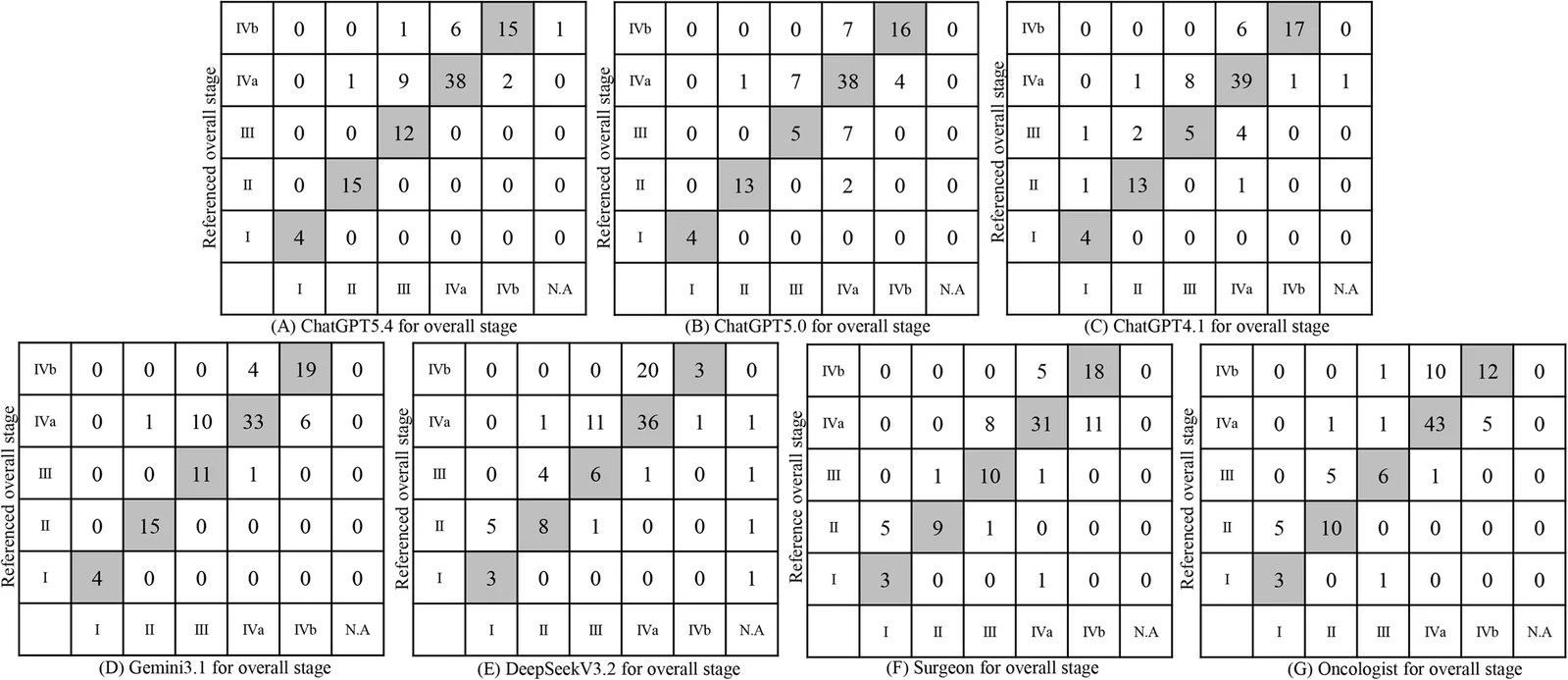
Confusion matrix for overall staging by LLMs (**A**–**E**) and human experts (**F**, **G**)

Confusion matrices of the LLMs for T-categorisation, N-categorisation and overall staging are shown in Figs. 3, 4 and 5, respectively. The accuracy of the LLMs for T-categorisation, N-categorisation and overall staging is shown in Table 3. The LLMs showed accuracy of 54.8–76.0% for T-categorisation, 63.5–88.5% for N-categorisation and 53.8–80.8% for overall staging (Table 3). Compared with DeepSeekV3.2, after adjusting the significant *p*-value threshold to < 0.0045 using the Bonferroni correction, three versions of the ChatGPT showed significantly higher accuracies for T-categorisation (all *p* < 0.001 to *p* = 0.001), N-categorisation (all *p* < 0.001) and overall staging (all *p* < 0.001) (Table 4), and Gemini3.1 showed significantly higher accuracies for N-categorisation (*p* < 0.001) and overall staging (*p* < 0.001), but not for T-categorisation (*p* = 0.07). No differences in the accuracy for T-categorisation (*p* = 0.08 to > 0.99), N-categorisation (*p* = 0.45 to > 0.99) and overall staging (*p* = 0.15 to 0.82) between ChatGPT5.4, ChatGPT5.0, ChatGPT4.1 and Gemini3.1 (Table 4). Of note, clinical T4a category was mistakenly updated to T4b category in 2, 2, 1, 4 and 1 patients by ChatGPT5.4, ChatGPT5.0, ChatGPT4.1, Gemini3.1 and DeepSeekV3.2, respectively; and clinical T4b category was mistakenly downstaged in 7, 6, 6, 4 and 13 patients by ChatGPT5.4, ChatGPT5.0, ChatGPT4.1, Gemini3.1 and DeepSeekV3.2, respectively.

**Table 3:** Accuracy of the commercial LLMs and human expert for OCC staging based on the 104 descriptive MRI reports

	LLMs					Human experts	
	ChatGPT5.4	ChatGPT5.0	ChatGPT4.1	Gemini3.1	DeepSeekV3.2	Oral surgeon	Oncologist
T-categorisation							
No. of accurate categorisation	79	74	73	71	57	80	77
Accuracy (95% CI)	76.0% (67.6–84.3%)	71.2% (62.3–80.0%)	70.2% (61.3–79.1%)	68.3% (59.2–77.4%)	54.8% (45.1–64.5%)	76.9% (68.7–85.2%)	74.0% (65.5–82.6%)
N-categorisation							
No. of accurate categorisation	91	89	89	92	66	80	81
Accuracy (95% CI)	87.5% (81.0–94.0%)	85.6% (78.7–92.4%)	85.6% (78.7–92.4%)	88.5% (82.2–94.7%)	63.5% (54.1–72.9%)	76.9% (68.7–85.2%)	77.9% (69.8–86.0%)
Overall stage							
No. of accurate categorisation	84	76	78	82	56	71	74
Accuracy (95% CI)	80.8% (73.1–88.5%)	73.1% (64.4–81.7%)	75.0% (66.5–83.5%)	78.8% (70.9–86.8%)	53.8% (44.1–63.6%)	68.3% (59.2–77.4%)	71.2% (62.3–80.0%)

**Table 4:** Comparison in accuracy of the commercialised LLMs for cancer staging based on the pre-treatment MRI reports

	ChatGPT5.4	ChatGPT5.0	ChatGPT4.1	Gemini3.1	DeepSeekV3.2
T-categorisation					
ChatGPT5.4	-	0.44	0.33	0.08*	**< 0.001**
ChatGPT5.0	-	-	> 0.99*	0.74	**< 0.001**
ChatGPT4.1	-	-	-	0.88	**0.001**
Gemini3.1		-	-	-	0.07
N-categorisation					
ChatGPT5.4	-	0.73*	0.77*	> 0.99*	**< 0.001**
ChatGPT5.0	-	-	> 0.99*	0.45*	**< 0.001**
ChatGPT4.1	-	-	-	0.58*	**< 0.001**
Gemini3.1	-	-	-	**-**	**< 0.001**
Overall staging					
ChatGPT5.4	-	0.15*	0.29*	0.77*	**< 0.001**
ChatGPT5.0	-	-	0.82*	0.26*	**< 0.001**
ChatGPT4.1	-	-	-	0.56	**< 0.001**
Gemini3.1	-	-	-	**-**	**< 0.001**

Due to multiple comparisons in accuracies of each categorisation among different LLM models (*n* = 11 times) and based on the Bonferroni correction, only a comparison with a *p*-value of < 0.0045 was defined as statistically significant

Bold indicates statistically significant

* *p*-value was evaluated by using exact McNemar test

Results of the Inter-observer agreements for cancer staging between (1) the reference standard and the LLMs and (2) between two rounds of the testing for each of the LLMs are shown in the supplementary file. The macro-averaged F1-score, per-class precision and recall of the LLMs for T-categorisation, N-categorisation and overall staging are shown in Supplementary Tables 3, 4 and 5, respectively.

### Comparison of accuracy for cancer staging between the LLM and human experts

Confusion matrices of the human experts for T-categorisation, N-categorisation and overall staging are shown in Figs. 3, 4 and 5, respectively. Human experts showed accuracy of 74.0–76.9% for T-categorisation, 76.9–77.9% for N-categorisation and 68.3–71.2% for overall staging (Table 3). Compared with human experts, after adjusting significant *p*-value to < 0.01 using the Bonferroni correction, ChatGPT and Gemini3.1 showed similar accuracy for T-categorisation (*p* = 0.22 to > 0.99), N-categorisation (*p* = 0.02 to 0.14) and overall staging (*p* = 0.03 to 0.87) (Table 5); and DeepSeekV3.2 showed significantly lower accuracy for T-categorisation (*p* < 0.01) and overall staging (only *p* < 0.01 compared with oncologist), but not for N-categorisation (*p* = 0.01 to 0.02) (Table 5).

**Table 5:** Comparison of the accuracy of two human experts and LLMs for cancer staging based on the pre-treatment MRI reports

	ChatGPT5.4	ChatGPT5.0	ChatGPT4.1	Gemini3.1	DeepSeekV3.2
T-categorisation					
Oral surgeon	> 0.99	0.44	0.35	0.22	**< 0.01**
Oncologist	0.88	0.74	0.62	0.48	**< 0.01**
N-categorisation					
Oral surgeon	0.04	0.11	0.14	0.02*	0.02
Oncologist	0.04*	0.13*	0.13*	0.04	0.01
Overall staging					
Oral surgeon	0.03	0.51	0.32	0.07	0.04
Oncologist	0.12	0.87	0.62	0.24	**< 0.01**

Due to multiple comparisons in accuracies of each categorisation between human experts and five LLMs (*n* = 5) and based on the Bonferroni correction, only a comparison with a *p*-value of < 0.01 was defined as statistically significant

Bold indicates statistically significant

* *p*-value was evaluated by using exact McNemar test

## Discussion

In this study, we investigated the value of commercial LLMs, ChatGPT (version 5.4, 5.0 and 4.1), Gemini3.1 and DeepSeekV3.2, for OCC staging based on pre-treatment MRI reports that only contained sufficient descriptive content for this task. The LLMs showed accuracy of 54.8–76.0% for T-categorisation, 63.5–88.5% for N-categorisation and 53.8–80.8% for overall staging. Macro-averaged F1-scores showed similar trends to the accuracies for cancer staging using LLMs, and per-class analysis showed various ranges for precision and recall across cancer staging. Compared with DeepSeekV3.2, three ChatGPT versions and Gemini3.1 showed higher staging accuracy, except for Gemini3.1 in T-categorisation. No significant accuracy differences between ChatGPT and Gemini. We also compared the accuracy of the LLMs and two human experts from MDT for OCC staging. Results showed that the LLMs did not consistently outperform the MDT members in cancer staging based solely on the descriptive MRI reports.

Previous studies evaluating commercial LLMs for cancer staging from radiology reports showed variable T- and N-categorisation accuracies [14–16, 20–23, 45], possibly due to differences in the studied LLM models, cancer type, report type, prompt design and reference standard. Three head and neck cancer studies [15, 16, 23] using ChatGPT4, DeepSeekV3 and Claude3.7 Sonnet reported an accuracy of 0.73 to 0.95 for T-categorisation and 0.80 to 0.94 for N-categorisation, with Gupta et al [23] further showing structured prompting further improved accuracy from 0.73 to 0.81 for T-categorisation and from 0.80 to 0.83 for N-categorisation. In this study, we expected LLMs provided with descriptive reports to stage cancers according to the latest manual. However, we observed that the interim update in 2020 was inconsistently evaluated by the LLMs, thus potentially contributing to their low accuracy for T-categorisation in OCC. Nevertheless, three models (ChatGPT5.0, ChatGPT4.0 and Gemini3.1) were able to consistently report the referenced staging manual (8th edition) in each output report, whereas DeepSeekV3.2 and ChatGPT5.4 omitted this in 7/104 and 19/104 reports, respectively. Although ChatGPT5.4 was the latest version available during our study and expected to yield the best staging performance, it unexpectedly did not outperform ChatGPT5.0 or ChatGPT4.1, indicating that commercial LLMs may not be suitable for this task and additional dedicated healthcare LLMs are being encouraged [46, 47].

In patients with OCC, pre-treatment clinical cancer staging plays an important role in disease management, particularly in the decision of primary treatment strategy. While T1–T4a tumours are highly operable and typically treated with surgery with or without neck dissection, many extensive T4b tumours necessitate a shift to non-surgical palliative care. In this study, we found that the five tested LLMs misclassified 7 to 14 patients within the T4a and T4b categories, with DeepSeekV3.2 exhibiting the highest error rate. These findings suggest that relying on current LLMs for clinical staging based on the MRI report could mislead the MDT members to make inappropriate treatment decisions. Furthermore, LLMs can also produce outputs that are factually incorrect, nonsensical or fabricated, which is a phenomenon known as hallucination, being widely reported in radiology studies [7, 8, 15, 48–50]. In our study, DeepSeekV3.2 produced a hallucinated output for one input report. This hallucinated output provided T- and N-categories and overall stage but incorrectly classified the cancer as N3c, a category that does not exist in the referenced manual [42–44]. Such hallucinations also have the potential to mislead LLM users, especially those who lack relevant knowledge.

Oral surgeons and oncologists play an important role in the MDT for OCC management. This study additionally reported the accuracy of the cancer staging by the oral surgeon and oncologist. Results showed that the oral surgeon and oncologist had similar accuracy of 0.70 to 0.75 for cancer staging based on the MRI reports. Descriptive MRI reports commonly include a large number of radiological terms and expression types. Interpretation of these contents by oral surgeons and oncologists could be different based on their working experiences and professional specialties. Furthermore, the cancer staging manual for OCC was later updated [43] after the initial publication of the 8th edition manuals in 2017 [42, 44], which also may lead to confusion when being used in clinical practice by different professional experts. Therefore, structured MRI reports that include clinical cancer staging are highly recommended as they could bring the radiologist’s professional perspective to image interpretation to greatly assist in the multidisciplinary assessment of cancer staging. This ensures alignment among MDT members and coordinates their approach, leading to optimal patient treatment and consistent clinical decision-making.

It is worth noting that while this study was based on a single-centre dataset, the performance differences between LLMs and human experts remained robust enough to withstand strict Bonferroni corrections for *p*-value. Furthermore, our findings showed that even when inputting highly detailed descriptive MRI reports, the tested LLMs did not perform well enough to warrant clinical implementation. Applying these models to routine clinical reports of varying quality from multiple centres would likely further degrade performance. Additionally, while our cohort had an uneven disease stage distribution, the predominance of advanced-stage OCC cases represented the exact clinical scenario where LLM-assisted staging would theoretically be most valuable. However, the suboptimal performances of the tested LLMs in this cohort showed that they are not yet reliable enough for providing clinical staging standalone based on descriptive MRI reports.

This study has limitations. First, there are many LLMs but were only able to study five commercial LLMs from three companies, which were accessible to our intuition, and we focused only on one group of HNCs. Second, versions of both cancer staging criteria and LLMs are updated periodically, so results from the current study may be time sensitive. Third, this study did not clarify to human experts whether any references to the cancer staging manual are allowed and did not impose any time constraint. To do so, although it might result in an inequivalent comparison from a research perspective, as LLMs and human experts may have different levels of expertise in clinical cancer staging for OCC, we expected that this could take into account the scenario that commonly occurs in clinical practice, and so the results from this study could bring clinically impactful information to readers. Furthermore, this study only focused on MRI report–based clinical staging, which is only a part of the clinical staging in disease management by MDT for patients with OCC. In addition, since this study was conducted retrospectively, we were unable to evaluate the potential influences of the LLMs on the clinical workflow, such as reporting time, MDT  efficiency, clinician satisfaction and user acceptance.

## Conclusion

Our study investigated the role of commercial LLMs (ChatGPT5.4, ChatGPT5.0, ChatGPT4.1, Gemini3.1 and DeepSeekV3.2) for assigning T- and N-categories and overall stage for OCC based on descriptive MRI reports. Results showed that ChatGPT and Gemini3.1 outperformed DeepSeekV3.2 for assigning cancer staging. Furthermore, the tested LLMs did not consistently outperform MDT members. Although some of these tested LLMs achieved higher accuracy, overall performance and occasional hallucinated outputs indicated that they are not suitable as standalone staging tools. Moreover, the low accuracy of two MDT members in assigning cancer staging underscored the need for structured MRI reports that include clinical cancer staging. Such reports could potentially facilitate a collaborative multidisciplinary review of both images and text that ultimately ensures consistent clinical decision-making across patients.

## Supplementary information

ELECTRONIC SUPPLEMENTARY MATERIAL

## Data Availability

Data and materials will be available once requested.

## Abbreviations

AI: Artificial intelligence
AJCC: American Joint Committee on Cancer
CT: Computed tomography
HNC: Head and neck
LLM: Large language model
MDT: Multidisciplinary team
MRI: Magnetic resonance imaging
OCC: Oral cavity cancer
UICC: Union of International Cancer Committee
