# Supplementary material for: Commercial large language models for oral cavity cancer staging using descriptive pre-treatment MRI reports: ready for standalone use in clinical practice?
Source: Insights Imaging. 2026 Sep 21;17:233. doi: 10.1186/s13244-026-02409-y (PMC13594034; doi:10.1186/s13244-026-02409-y)
Supplement: Supplementary file 1 [file 13244_2026_2409_MOESM1_ESM.pdf]

Commercial large language models for oral cavity cancer staging using descriptive pre-treatment MRI reports: ready for standalone use in clinical practice?

ELECTRONIC SUPPLEMENTARY MATERIAL

Supplementary methods

<u>Inclusion and exclusion criteria for selecting MRI reports</u>

MRI reports that fulfilled the following inclusion criteria were included in the study: (1) adult patients with a new previously untreated biopsy-proven squamous cell carcinoma of the oral cavity, including the oral tongue, retromolar trigone, floor of mouth, gingiva, or hard palate; and (2) patients who had pre-treatment staging head and neck MRI performed and reported between January 2015 and April 2025 at the Prince of Wales Hospital, Hong Kong, S.A.R. and (3) the MRI reports were retrievable.

Exclusion criteria were: (1) patient who had a history of OCC, or (2) the radiology reports did not include sufficient descriptive information, which is shown below for cancer staging.

<u>Pre-treatment MRI reports acquisition and eligibility analysis</u>

The full pre-treatment head and neck MRI reports from patients who fulfilled the inclusion criteria were extracted from the Radiology Information System (RIS) for analysis. The eligibility of the MRI reports was evaluated by two on-board certified radiologists subspecialising in head and neck radiology over 5 years (H.S.L. and H.M.K.) and a researcher with over 12-year of experience in head and neck radiology (Q.Y.H.A). Eligible MRI reports must include sufficient descriptive information for cancer staging, which included location, size, depth of invasion, and invasion extent of the primary tumour; and location, size, latelarity, and numbers of the nodal metastatses. A lymph node without description of radiological extranodal

extension (ENE) was regarded as clinically negative ENE and radiologically overt ENE were regarded as clinically positive ENE. Contents with a concern for patient identifiers were excluded from all of the descriptive reports for further assessments by LLM and human experts.

Procedures of the commercial LLMs to process the descriptive OCC MRI reports

This study used website access platform via The University of Hong Kong to all the LLMs models with defaulted setting. Because web interfaces use proprietary, dynamically optimised default settings that cannot be adjusted by the user, the default system configurations of the respective platforms as of between July 2025 and March 2026 were used. No custom system instructions or advanced user settings were modified. No additional information was provided to the models, or no additional setting was performed to the models to infer the internal knowledge. Descriptive content of the primary tumour and regional nodal metastases from each MRI report was orderly input to those three commercial LLMs, respectively with the prompt of "Staging the case based on the following descriptive MRI content provided". A new chat box was initiated on the LLM platforms for uploading each of the reports and none of the reports were retained for LLM modal refinement. The output from each time of the input was generated once and each output was collected for further analysis.

Other matrices for evaluating performance

The macro-averaged F1-score, per-class precision and recall of the LLMs for cancer staging were calculated based on the following equations:

1. Macro F1-Score

The macro F1-score = mean of per-class F1-scores across all *K* classes:

$$\mathrm{MacroF1} = \frac{1}{K} \cdot \sum_{c \in K} F1_{\mathrm{c}}$$

2. Per-Class F1-Score

The F1-score for class *c* is the harmonic mean of $\mathrm{Precision}_c$ and Recall_c:

$$F1_c = 2 \times \frac{\text{Precision}_c \times \text{Recall}_c}{\text{Precision}_c + \text{Recall}_c}$$

This is equivalent to:

$$F1_c = \frac{2 \cdot TP_c}{(2 \cdot TP_c + FP_c + FN_c)}$$

3. Per-Class Precision

Precision (specificity [Sp]) for class *c* measures the fraction of predicted positives that are truly positive:

$$\text{Precision}_c = \text{Sp} = \frac{TP_c}{TP_c + FP_c}$$

4. Per-Class Recall

Recall (sensitivity [Se]) for class *c* measures the fraction of true positives that are correctly identified:

$$\text{Recall}_c = \text{Se} = \frac{TP_c}{TP_c + FN_c}$$

Notation

Let C be the set of classes with $|C| = K$. For a given class $c \in C$, define:

TP_c = True Positives for class *c*

FP_c = False Positives for class *c*

FN_c = False Negatives for class *c*

Supplementary results

Inter-observer agreements in cancer staging

LLMs

The weighted kappa tests showed that the inter-observer agreements in T-categorisation, N-categorisation and overall staging between the reference standard and LLMs ranged from 0.62 to 0.78, 0.61 to 0.88, and 0.57 to 0.79, respectively, of which ChatGPT5.4 and DeepSeekV3.2 showed the highest and lower weighted kappa coefficients, respectively (Supplementary Table 2). The agreements of the LLMs for T-categorisation, N-categorisation and overall staging between two rounds of the tests showed a weighted kappa coefficient of 0.891, 0.910 and 0.864, respectively in ChatGPT5.4; of 0.919, 0.901, and 0.872, respectively in ChatGPT5.0; of 0.849, 0.862 and 0.782, respectively in ChatGPT4.1; of 0.886, 0.897, and 0.842, respectively in Gemini3.1, and of 0.740, 0.820 and 0.588, respectively in DeepSeekV3.2.

Human experts

The weighted kappa coefficients for inter-observer agreement in T-categorisation, N-categorisation and overall staging between the reference standard and human experts ranged from 0.77 - 0.82, 0.79 – 0.79, and 0.72 – 0.73, respectively (Supplementary Table 2), and between two human experts was 0.782, 0.691 and 0.711 respectively.

Supplementary tables

Supplementary Table 1. Clinical TNM cancer staging for oral cavity cancer based on the eighth edition of the AJCC Cancer Staging Manual and the following refinement published in 2020 (1,2)

**T-category**	
T1	Tumour ≤ 2 cm with DOI ≤ 5 mm
T2	Tumour ≤2 cm with DOI >5 mm or Tumour > 2 cm and ≤ 4 cm with DOI ≤ 10 mm
T3	Tumor >2 cm and ≤4 cm with DOI >10 mm or Tumor >4 cm with DOI ≤10 mm
T4a	Tumor >4 cm with DOI >10 mm or Tumor invades adjacent structures only (e.g., through cortical bone of mandible or maxilla, or involves the maxillary sinus or skin of the face)
T4b	Tumor invades masticator space, pterygoid plates, or skull base and/or encases the internal carotid artery
**N-category**	
N0	No regional lymph node metastasis
N1	Metastasis in a single ipsilateral lymph node, ≤ 3 cm in greatest dimension and clinical ENE(−)
N2a	Metastasis in a single ipsilateral node > 3 cm but not > 6 cm in greatest dimension and clinical ENE(−)
N2b	Metastases in multiple ipsilateral nodes, none > 6 cm in greatest dimension and clinical ENE(−)
N2c	Metastases in bilateral or contralateral lymph node(s), none > 6 cm in greatest dimension and clinical ENE(−)
N3a	Metastasis in a lymph node > 6 cm in greatest dimension and ENE(−)
N3b	Metastasis in any node(s) with clinically overt ENE(+) (cENE)
**M-category**	
M0	No evidence of metastases
M1	Distant metastases
**Overall stage**	
Stage I	T1N0M0
Stage II	T2N0M0
Stage III	T3N0M0 or T1-3N1M0
Stage IVa	T4aN0-1M0 or T1-4aN2M0
Stage IVb	T_{any}N3M0 or T4bN_{any}M0
Stage IVc	$T_{any}N_{any}$M1

DOI = depth of invasion (not tumour thickness), ENE = extranodal extension

Supplementary Table 2. Inter-observer agreement in T-categorisation, N-categorisation, and overall staging between the reference standard and LLMs and human experts

	Weighted kappa coefficients (95%CI)		
	T-category	N-category	Overall stage
**LLMs**			
ChatGPT5.4	0.78 (0.69 – 0.87)	0.88 (0.80 – 0.95)	0.79 (0.68 – 0.89)
ChatGPT5.0	0.74 (0.65 – 0.84)	0.83 (0.74 – 0.92)	0.73 (0.63 – 0.84)
ChatGPT4.1	0.74 (0.64 -0.83)	0.80 (0.70 – 0.91)	0.74 (0.64 – 0.84)
Gemini3.1	0.73 (0.64 – 0.82)	0.88 (0.81 – 0.95)	0.81 (0.74 – 0.89)
DeepSeekV3.2	0.62 (0.53 – 0.70)	0.61 (0.50 – 0.72)	0.57 (0.47 – 0.67)
**Human experts**			
Surgeon	0.82 (0.75 - -0.88)	0.79 (0.70 – 0.87)	0.72 (0.64 -0.81)
Oncologist	0.77 (0.69 – 0.85)	0.79 (0.70 – 0.88)	0.73 (0.65 – 0.82)

Supplementary Table 3. The averaged F1-score, per-class precision and recall for T-categorisation among LLMs

	ChatGPT5.4	ChatGPT5.0	ChatGPT4.1	Gemini3.1	DeepSeekV3.2
Macro-averaged F1-score	0.82 (0.75-0.88)	0.77 (0.70-0.84)	0.71(0.59-0.80)	0.77(0.70-0.83)	0.42 (0.32-0.56)
Per-class Precision					
Precision T1	0.98 (0.95-1.00)	0.98 (0.96-1.00)	0.66 (0.20-1.00)	0.98 (0.94-1.00)	0.30 (0.00-0.64)
Precision T2	1.00 (1.00-1.00)	1.00 (1.00-1.00)	0.81 (0.62-0.95)	1.00 (1.00-1.00)	0.61 (0.38-0.83)
Precision T3	0.55 (0.39-0.71)	0.53 (0.29-0.75)	0.50 (0.26-0.74)	0.46 (0.31-0.60)	0.40 (0.18-0.63)
Precision T4a	0.81 (0.66-0.94)	0.62 (0.48-0.75)	0.67 (0.53-0.80)	0.78 (0.56-0.95)	0.63 (0.49-0.76)
Precision T4b	0.85 (0.62-1.00)	0.86 (0.64-1.00)	0.92 (0.75-1.00)	0.78 (0.57-0.95)	0.83 (0.50-1.00)
Per-class Recall					
Recall T1	0.98 (0.95-1.00)	0.98 (0.96-1.00)	0.98 (0.96-1.00)	0.98 (0.96-1.00)	0.74 (0.00-1.00)
Recall T2	1.00 (1.00-1.00)	0.94 (0.81-1.00)	0.94 (0.81-1.00)	1.00 (1.00-1.00)	0.61 (0.38-0.84)
Recall T3	0.95 (0.85-1.00)	0.45 (0.24-0.67)	0.41 (0.20-0.62)	0.95 (0.85-1.00)	0.36 (0.17-0.58)
Recall T4a	0.60 (0.44-0.74)	0.74 (0.60-0.87)	0.74 (0.60-0.87)	0.33 (0.20-0.49)	0.72 (0.57-0.85)
Recall T4b	0.61 (0.38-0.83)	0.67 (0.44-0.88)	0.67 (0.43-0.88)	0.78 (0.57-0.95)	0.28 (0.08-0.50)

Supplementary Table 4. The averaged F1-score, per-class precision and recall for N-categorisation among LLMs

	ChatGPT5.4	ChatGPT5.0	ChatGPT4.1	Gemini3.1	DeepSeekV3.2
Macro-averaged F1-score	0.71 (0.61-0.87)	0.69 (0.60-0.85)	0.64 (0.54-0.85)	0.71(0.61-0.89)	0.39 (0.31-0.50)
Per-class Precision					
Precision N0	0.88 (0.78-0.96)	0.89 (0.80-0.98)	0.93 (0.85-1.00)	0.93 (0.85-1.00)	0.95 (0.87-1.00)
Precision N1	0.85 (0.50-1.00)	0.87 (0.57-1.00)	0.58 (0.29-0.88)	0.85 (0.50-1.00)	0.32 (0.15-0.50)
Precision N2a	0.00 (0.00-0.00)	0.00 (0.00-0.00)	0.00 (0.00-0.00)	0.00 (0.00-0.00)	0.00 (0.00-0.00)
Precision N2b	0.96 (0.86-1.00)	0.96 (0.86-1.00)	0.91 (0.28-1.00)	0.96 (0.87-1.00)	0.45 (0.23-0.67)
Precision N2c	0.86 (0.67-1.00)	0.84 (0.62-1.00)	0.84 (0.63-1.00)	0.92 (0.75-1.00)	0.90 (0.67-1.00)
Precision N3a	-	-	-	-	-
Precision N3b	0.86 (0.50-1.00)	0.64 (0.33-0.91)	0.89 (0.64-1.00)	0.58 (0.29-0.88)	0.63 (0.00-1.00)
Per-class Recall					
Recall N0	0.98 (0.93-1.00)	0.95 (0.88-1.00)	0.95 (0.88-1.00)	0.98 (0.93-1.00)	0.86 (0.76-0.96)
Recall N1	0.60 (0.25-0.90)	0.70 (0.38-1.00)	0.70 (0.38-1.00)	0.60 (0.25-0.90)	0.90 (0.67-1.00)
Recall N2a	0.00 (0.00-0.00)	0.00 (0.00-0.00)	0.00 (0.00-0.00)	0.00 (0.00-0.00)	0.00 (0.00-0.00)
Recall N2b	0.85 (0.70-0.97)	0.82 (0.65-0.96)	0.78 (0.61-0.93)	0.89 (0.76-1.00)	0.33 (0.16-0.52)
Recall N2c	0.93 (0.75-1.00)	0.79 (0.55-1.00)	0.79 (0.54-1.00)	0.86 (0.64-1.00)	0.64 (0.38-0.90)
Recall N3a	-	-	-	-	-
Recall N3b	0.75 (0.40-1.00)	0.88 (0.60-1.00)	1.00 (1.00-1.00)	0.88 (0.60-1.00)	0.12 (0.00-0.43)

Supplementary Table 5. The averaged F1-score, per-class precision and recall for overall stage among LLMs

	ChatGPT5.4	ChatGPT5.0	ChatGPT4.1	Gemini3.1	DeepSeekV3.2
Averaged F1-score	0.75 (0.65-0.89)	0.75 (0.67-0.83)	0.72(0.59-0.82)	0.83(0.77-0.89)	0.39 (0.28-0.49)
Per-class Precision					
Precision Stage I	0.98 (0.97-1.00)	0.98 (0.95-1.00)	0.66 (0.20-1.00)	0.98 (0.97-1.00)	0.38 (0.00-0.75)
Precision Stage II	0.94 (0.79-1.00)	0.93 (0.75-1.00)	0.81 (0.59-1.00)	0.94 (0.79-1.00)	0.61 (0.33-0.89)
Precision Stage III	0.55 (0.33-0.76)	0.42 (0.13-0.73)	0.39 (0.13-0.67)	0.53 (0.31-0.75)	0.33 (0.13-0.57)
Precision Stage IVa	0.86 (0.76-0.96)	0.70 (0.58-0.82)	0.78 (0.66-0.89)	0.87 (0.76-0.97)	0.63 (0.50-0.76)
Precision Stage IVb	0.88 (0.71-1.00)	0.80 (0.61-0.95)	0.89 (0.74-1.00)	0.76 (0.58-0.92)	0.74 (0.00-1.00)
Per-class Recall					
Recall Stage I	0.98 (0.97-1.00	0.98 (0.95-1.00)	0.98 (0.95-1.00)	0.98 (0.97-1.00)	0.74 (0.00-1.00)
Recall Stage II	1.00 (1.00-1.00)	0.87 (0.67-1.00)	0.87 (0.67-1.00)	1.00 (1.00-1.00)	0.53 (0.27-0.80)
Recall Stage III	1.00 (1.00-1.00)	0.42 (0.13-0.71)	0.42 (0.13-0.71)	0.92 (0.71-1.00)	0.50 (0.20-0.80)
Recall Stage IVa	0.76 (0.64-0.87)	0.76 (0.64-0.88)	0.78 (0.66-0.89)	0.66 (0.53-0.79)	0.72 (0.59-0.84)
Recall Stage IVb	0.65 (0.45-0.84)	0.70 (0.50-0.88)	0.74 (0.55-0.91	0.83 (0.65-0.96)	0.13 (0.00-0.29)

Supplementary references:

1. Amin MB, Edge SB, Greene FL, Byrd DR, Brookland RK, Washington MK, et al., editors. AJCC Cancer Staging Manual. 8th ed. New York NY: Springer; 2017.
2. Zanoni DK, Patel SG. New AJCC: How does it impact oral cancers? Oral Oncol 2020;104:104607. https://doi.org/10.1016/j.oraloncology.2020.104607
